# Closing the gender leadership gap: a multi-centre cross-country comparison of women in management and leadership in academic health centres in the European Union

**DOI:** 10.1186/s12960-016-0175-y

**Published:** 2017-01-06

**Authors:** Ellen Kuhlmann, Pavel V. Ovseiko, Christine Kurmeyer, Karin Gutiérrez-Lobos, Sandra Steinböck, Mia von Knorring, Alastair M. Buchan, Mats Brommels

**Affiliations:** 1 Medical Management Centre, LIME, Karolinska Institutet, Stockholm, Sweden; 2 Institute for Economics, Labour and Culture, Goethe-University Frankfurt, Frankfurt, Germany; 3 Medical Sciences Division, John Radcliffe Hospital, University of Oxford, Oxford, OX3 9DU United Kingdom; 4 Women and Equal Opportunities Office, Charité – Universitätsmedizin Berlin, Berlin, Germany; 5 Medical University of Vienna, Vienna, Austria

**Keywords:** Women in medicine, Women in science, Gender equality, Leadership, Medical management, Academic medicine, Academic health centres, European Union

## Abstract

**Background:**

Women’s participation in medicine and the need for gender equality in healthcare are increasingly recognised, yet little attention is paid to leadership and management positions in large publicly funded academic health centres. This study illustrates such a need, taking the case of four large European centres: Charité – Universitätsmedizin Berlin (Germany), Karolinska Institutet (Sweden), Medizinische Universität Wien (Austria), and Oxford Academic Health Science Centre (United Kingdom).

**Case:**

The percentage of female medical students and doctors in all four countries is now well within the 40–60% gender balance zone. Women are less well represented among specialists and remain significantly under-represented among senior doctors and full professors. All four centres have made progress in closing the gender leadership gap on boards and other top-level decision-making bodies, but a gender leadership gap remains relevant. The level of achieved gender balance varies significantly between the centres and largely mirrors country-specific welfare state models, with more equal gender relations in Sweden than in the other countries. Notably, there are also similar trends across countries and centres: gender inequality is stronger within academic enterprises than within hospital enterprises and stronger in middle management than at the top level. These novel findings reveal fissures in the ‘glass ceiling’ effects at top-level management, while the barriers for women shift to middle-level management and remain strong in academic positions. The uneven shifts in the leadership gap are highly relevant and have policy implications.

**Conclusion:**

Setting gender balance objectives exclusively for top-level decision-making bodies may not effectively promote a wider goal of gender equality. Academic health centres should pay greater attention to gender equality as an issue of organisational performance and good leadership at all levels of management, with particular attention to academic enterprises and newly created management structures. Developing comprehensive gender-sensitive health workforce monitoring systems and comparing progress across academic health centres in Europe could help to identify the gender leadership gap and utilise health human resources more effectively.

## Background

Women’s participation in medicine and the need for gender equality in healthcare are increasingly recognised, yet little attention is paid to leadership and management positions in large publicly funded academic health centres. Greater involvement of women in leadership and management is not only an issue of equality and human rights but also an important strategy towards effective utilisation of women’s qualifications, greater creativity and innovation, and improved organisational performance [1–3].

International evidence indicates persisting gender inequality and under-utilisation of women’s expertise in leadership and management positions in academic medicine. For example, in the United States, 47% of medical school matriculants but only 21% of full professors, 15% of permanent department chairs, and 16% of medical school deans are women [4]. In Canada, the first female dean of a faculty of medicine was only appointed in 1999 [5] and there is still just one female dean of a total of 17 faculties of medicine [6]. In Australia, women make up more than half of medical graduates but only 28% of medical school deans, 29% of governing board or committee members of medical colleges, and 12.5% of chief executives of hospitals larger than 1000 beds [7]. In the United Kingdom, only two out of ten chief executives of the largest teaching hospitals are women [8]. While gender equality has moved higher up on the European Union (EU) policy agenda in recent decades [1, 9], the implementation of policies is more difficult and diverse and poorly monitored [10]. Comparative data for EU academic health centres are not available, and national data are scattered and lack standardisation.

This study sets out to critically explore and compare the representation of women in leadership and management in European academic health centres. As systematic data are lacking, we—academic and clinical leaders, researchers, and gender equality practitioners from *Charité – Universitätsmedizin Berlin* (Berlin, Germany), *Karolinska Institutet* (Stockholm, Sweden), *Medizinische Universität Wien* (Vienna, Austria), and Oxford Academic Health Science Centre (Oxford, United Kingdom)—use our own institutions as case studies. Throughout this article, we refer to them collectively as ‘academic health centres’.

Similarities between the four centres and different country-specific contexts make multi-centre cross-national comparison an important tool to reveal general trends and factors affecting gender equality, yet a framework for comparison is lacking. To take a first step towards developing a common analytical framework and indicators for data collection and analysis, we held a half-day workshop in October 2015 at Charité. Following a bottom-up and context-sensitive approach, we started with describing the management structure of each of the four centres and explaining and translating formal management and leadership positions into English. Next, we explored similarities and differences and tried to identify categories and indicators for comparison. On the backdrop of numerous differences, we decided to reduce the complexity of management structure to three levels (top, middle, and lower) and to use these levels for comparison. The top level includes the boards and senior leaders, while ‘middle’ refers to large units and ‘low’ to smaller units. Then, we jointly assigned the management positions within the four individual cases to these three levels and refined the assessment framework (presented in Table 1).

**Table 1:** Gender breakdown of key leaders and managers at Charité, Karolinska Institutet, Oxford, and Vienna academic health centres, May 2016 (or latest available)

Centre/enterprise	Top level (boards)	Middle-level (large units)	Lower-level (smaller units)
Charité (Germany)	Supervisory board: chair F, 6 M/6 F (>50% F) Executive board: chair M, 3 M/1 F (25% F)	Directors of centres: 13 M/4 F (24% F)	Directors of clinics/institutes: 82 M/23 F (22% F)
Hospital	Senior management team: CEO F, 2 M/2 F (50% F)		
University^a^	Faculty board: dean M, 4 M/1 F (20% F)		
Karolinska Institutet (Sweden) Hospital	Board of directors: chair M, 6 M/5 F (45% F) Management team: CEO M, 9 M/6 F (40% F)	Chiefs of divisions: 5 M/2 F (29%)	Heads of departments: 36 M/39 F (52% F)
University	Board: chair M, 9 M/12 F (57% F) Management team: vice-chancellor F, 3 M/3 F (50% F)	Heads of departments: 16 M/6 F (27% F)	Variety of organisational structure; data not available
Oxford (United Kingdom)	AHSC board: chair M, 4 M/1 F (20% F)		
Hospital^b^	Board of directors: chair F, CEO M, 12 M/4 F (25% F)	Directors of divisions: 5 M/0 F (0% F)	Clinical directors: 11 M/7 F (39% F)
University	Chancellor: *M* Council: vice-chancellor *F*, 18 M/8 F (31% F) Medical sciences board: dean M, 14 M/7 F (33% F)	Medical sciences heads of departments: 15 M/2 F (12% F)	Medical sciences directors of research institutes/centres/units: 18 M/7 F (28% F)
Vienna (Austria)	Supervisory board: chair M, 4 M (0% F) Management board: 2 M (0% F)	Heads of clinics, clinical institutes, centres, and special institutions: 35 M/8 F (19% F)	Heads of departments and divisions: 67 M/22 F (25% F)
Hospital	Directors: CEO M, 2 M/3 F (60% F)		
University	Senate: chair M, 11 M/16 F (59% F) Council: chair M, 3 M/2 F (40% F) Rectorate: rector M, 3 M/2 F (40% F)		

Source: own calculations based on information from HR Officers, websites, and reports

*M* male, *F* female, *N/A* not available, *AHSC* Academic Health Science Centre

^a^Single medical faculty serving *Humboldt Universtität zu Berlin* and *Freie Universität Berlin*

^b^Oxford University Hospitals National Health Service Trust

After the workshop, we gathered material from published reports, websites, and institutional human resources and/or equal opportunity officers. We analysed sex-disaggregated quantitative data on the representation of women in leadership and management and qualitative information on actions to advance gender equality in our centres. Our approach and findings are discussed below. Notwithstanding the salience of country-specific contexts, we believe that our mapping exercise may be relevant to other academic health centres wishing to benchmark the representation of women in leadership and management in their centres and take action to reduce the gender leadership gap, thus promoting a more effective use of human resources.

## Case presentation

Our institutions are large academic health centres characterised by a shared commitment to the integration of university and hospital missions of research, education, and patient care [11, 12]. They all have established gender equality policies, yet there are differences in the models of academic-clinical integration, in the types of healthcare systems, and in the country-specific levels of gender equality.

- Models of academic-clinical integration range from a single corporation uniting hospital and university missions at Charité, to jointly governed hospital and university missions of separate organisations at Vienna, and aligned separate university and hospital organisations at Oxford and Karolinska Institutet.
- Healthcare systems range from a centralised National Health Service in England/United Kingdom, to a decentralised universal system in Sweden, and a federalist corporatist system in Germany and Austria.
- Gender equality—measured by the Gender Equality Index (on a scale from 1/lowest to 100/highest) across eight domains including work, money, knowledge, time, power, health, violence, and intersecting inequalities—ranges from high in Sweden (74.2) to medium in the United Kingdom (58.0), Germany (55.3), and Austria (50.2) [13]. In the European Union, the Gender Equality Index averages 52.9, ranging between 33.7 in Romania and 74.2 in Sweden [13].

### Women in leadership and management: where are we now?

**Fig. 1:**
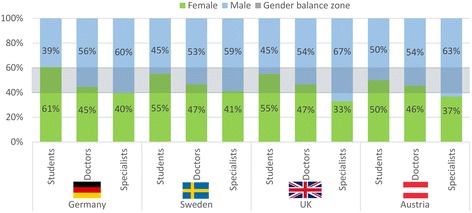
Gender breakdown of medical students, doctors, and specialists in Germany, Sweden, England, and Austria, 2014 (or nearest year). Source: OECD [15], *Statistisches Bundesamt* [34], *Bundesärztekammer* [35], *Socialstyrelsen* [36], Medical Schools Council [37], General Medical Council [38], *Österreichische Ärztekammer*, and Statistik Austria [39]

The current gender breakdown of medical students, doctors, and specialists in Germany, Sweden, United Kingdom, and Austria is compared in Fig. 1. The percentage of female medical students in all four countries exceeds a 40% threshold for gender balance proposed by the European Commission [14]. In Germany, the pendulum might even be swinging towards an over-representation of female students [15]. The percentages of female doctors in all four countries remains lower than those in the group of medical students, reflecting lower percentages of female medical students in previous decades, but it is well within the 40–60% gender balance zone and similar in the four countries (45–47%). The situation changes when looking at the group of specialists, where the time lag does not hold as a sole argument and country differences are becoming more relevant (33–41%). Women have only marginally achieved gender balance in specialist careers in Germany and Sweden and remain narrowly under-represented in Austria and more so in the United Kingdom.

**Fig. 2:**
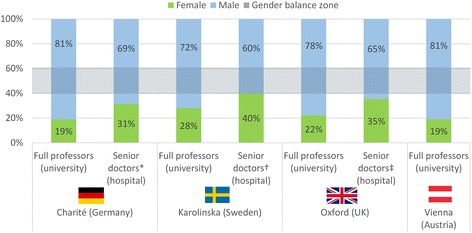
Gender breakdown of full professors and senior doctors at Charité, Karolinska Institutet, Oxford, and Vienna academic health centres, 2015. Source: own calculations based on information from HR/equal opportunity officers, documents, and reports. Note: the category of senior doctors serves as a proxy for a rough comparison, as there are no equivalent positions in the four centres/countries; at Charité (*Oberärztinnen und -ärzte) and Karolinska Hospital (†*overläkare*), a comparable category of an appointed position of doctors with leadership and usually also some management responsibilities is available, while this category (‡consultants) is different and based on a job position at the Oxford University Hospitals National Health Service Trust; data for Vienna are not available

Although medicine in the EU has largely become a gender-balanced profession at the junior level, in all four academic health centres but Karolinska Institutet, women remain under-represented among senior doctors (Fig. 2). No exact data are available for Vienna [16], but the national figure of 37% women specialist doctors (Fig. 1) (as a basic requirement for becoming a senior doctor) supports our argument. Remarkably, gender imbalance is greater within the university enterprise than within the hospital enterprise; women remain under-represented in top academic ranks in all four academic health centres but to a lesser extent at Karolinska Institutet (Fig. 2).

The under-representation of women among full professors and senior doctors creates a leadership gap in the organisational units but in different and uneven ways (Table 1). For example, at Oxford, women are virtually not represented as the leaders of large organisational units (middle management) either in the university or hospital enterprise but they are relatively well represented as the leaders of smaller organisational (lower-level management) units in both academic and clinical enterprises. Across all four centres, the gender leadership gap tends to be narrower within the hospital than within the academic enterprise and may even achieve a balance. For instance, women represent 52% of the heads of department at Karolinska Hospital. However, Karolinska Hospital is now in the process of a major reorganisation where new managers are being appointed to new positions, and it is currently not clear whether and how this will affect the achieved gender balance. Developments in Vienna reveal a remaking of inequality in the process of organisational restructuring and new top-level management boards, despite clear quotas and well-achieved gender balance. In all centres except Charité, the gender leadership gap is narrower in lower-level organisational units.

The findings indicate important shifts in the gender leadership gap, which challenge the focus of gender policies and research on top-level positions. Remarkably, hospital management now seems to be more permeable for women than academic management. Although changes in gender equality are driven by many different factors [10, 17–22], our findings call for a closer look at the effects of the new modes of hospital governance and clinical management [23] on gender equality. Hospital governance has moved decision-making powers to the level of organisational units and has engaged doctors in management more closely [24]. These developments might affect gender equality and, therefore, need to be monitored and researched more carefully.

### Action to advance gender equality in the medical workforce

Notwithstanding differences in governance and management arrangements as well as in gender equality policies, all centres have taken action to improve gender equality. Major tools include equal opportunity recruitment, anti-discriminatory practices, monitoring of gender equality, career development workshops and seminars, gender-sensitive appointment and promotion criteria, support for parents and carers, flexible and part-time working arrangements, mentoring programmes and networking for women, unconscious bias and diversity training for all staff, and inclusion of gender issues in teaching curricula [25]. While these tools are broadly similar and applied, at least to some extent, at all four centres, the socio-legal contexts and governance frameworks vary.

To begin with, the Federal State of Berlin makes legal provisions for the position of Central Representatives for Equal Opportunities, and Charité has established a comprehensive policy and action plan [26]. The Central Representative for Equal Opportunities is an elected, and thus more independent, position, which is affiliated with the top-level executive (as an advisory member on Charité’s Supervisory Board with limited decision-making rights). The Central Representative is supported by three elected deputies and by secretarial capacities. There are no compulsory quotas, but a set of voluntary targets for achieving a greater gender balance at different organisational levels has been agreed. Gender equality is well monitored, notably also on the levels of institutes and centres, and a comprehensive bi-annual gender equality report to the Berlin Senate is mandatory [27]. Recently, gender equality objectives have been linked to performance-related pay (10% of the salary) in order to provide leaders and managers with financial incentives to achieve gender equality objectives. Plans are currently under discussion to introduce a quota of 28% female professors for new appointments.

Karolinska Institutet has established a Council for Equal Treatment at the central level and Equal Treatment representatives at the level of departments and developed a comprehensive action plan for equal treatment, which is periodically revised [28, 29]. No compulsory quotas exist, but both the Karolinska Hospital and Karolinska Institutet have set (and periodically revised) clear targets for female leadership, which are closely monitored and supported by performance incentives and voluntary action. One important tool is to increase the share of women among newly recruited professors. A recent goal is set to achieve 47% female professors, which is supported by short-term action, such as monetary incentives at the department level for the appointment of a women professor [29]. Long-term activities include, for instance, ‘providing funds for excellent female researchers to increase the time for research’ ([30], p. 11).

At Oxford and elsewhere in the United Kingdom, the Athena Project and the Scientific Women’s Academic Network (Athena SWAN) Charter for Women in Science provides a common framework for advancing gender equality in science and medicine. Every university medical sciences department and the Medical Sciences Division as a whole have Athena SWAN committees and coordinators who develop and implement action plans. The Charter recognises the efforts of participating institutions with institutional and departmental bronze, silver, and gold awards [31]. The Charter has become particularly influential since 2011, when the government made the achievement of at least a silver award a precondition for participation in the competition for translational research funding provided by the National Institute for Health Research (NIHR) [32]. Awards take into account different starting conditions in different disciplines and institutions and focus on the development and implementation of action plans to remove structural and cultural gender-based barriers to equal participation rather than the achievement of immediate targets or quotas.

At Vienna Medical University, according to the Austrian Universities Act of 2002, there is a compulsory quota of 40% women on university boards and decision-making committees. This quota is monitored and enforced by the Federal Ministry for Science, Research and Economy on a yearly basis [33]. Moreover, Vienna Medical University has carried out a gender-sensitive assessment of the entrance exams for women and men in medicine and established a new model, which avoids gender-discriminating test methods. The Gender Mainstreaming Office develops and implements measures to support women’s advancement and to monitor internal data regarding compliance with the quota. The Working Group for Equal Opportunities in the university and an anti-discrimination group in the hospital further contribute to the development and implementation of gender equality measures in the context of wider equality measures.

Overall, our case study material illustrates important steps taken at all centres to reduce the gender leadership gap. Although policy frameworks and action plans are usually broad and stretch across organisational levels, gender equality policies focus on top-level positions, especially appointment of professors. In all centres, this is complemented with a range of activities on the individual level such as mentoring programmes for women, and training seminars and workshops to improve the gender-sensitivity of managers. Less attention has been paid so far to the improvement of organisational performance and management, but recent connections between gender equality goals and performance incentives at the level of departments and institutes (middle management) might indicate a move in this direction. There are also no specific education programmes for gender-sensitive clinical leadership, such as modules in Masters’ and Ph.D. courses.

In the course of data collection for our comparison, we found that gender-disaggregated data are not systematically collected from a management approach, although each of our centres has well-established organisational structures for human resources and equality. Such data are especially poor for middle and lower organisational levels. This is alarming, because academic health centres with devolved and decentralised organisational structures make important health human resources and funding decisions precisely at these levels. We also found a lack of standardised indicators and efforts to compare the levels of gender equality in academic health centres across the EU member states.

## Conclusions

In this multi-centre cross-country comparison, we set out to explore a gender gap in leadership and management positions in large publicly funded academic health centres, taking four centres in the EU as case studies. While approximately half of current medical students are women, significantly fewer women than men hold leadership and management positions within academic health centres. Two important patterns can be identified across our centres and countries. First, gender inequality is strongest at the middle level of leadership and management as well as among full professors. Second, the gender leadership gap tends to be narrower within the hospital than within the academic enterprise. Also of importance is that new forms of management (e.g. mergers, privatisation, or new performance management schemes) may embody new risks for gender equality and therefore need careful gender assessment. These findings reveal novel and uneven shifts in the gender leadership gap towards the less-well-monitored middle-level management positions.

The findings suggest that clear targets and action plans to increase the representation of women in top-level decision-making positions are important but not sufficient. A focus on top-level decision-making bodies is not sustainable, because the inequality gap may move down to middle-management positions that are less well monitored. Furthermore, stronger inequality in the academic enterprise is especially alarming, because it compromises commitment to high-quality medical education and negatively effects the future generation of doctors. Action should be taken to assess the effects of changing clinical management through the lens of gender and to develop comprehensive gender-monitoring systems based on standardised indicators, which enable comparison across academic health centres. This could help to identify and close the gender leadership gap and to utilise health human resources more effectively.

## Abbreviations

Athena SWAN: Athena Project and the Scientific Women’s Academic Network
EU: European Union
NIHR: National Institute for Health Research
